# Health care experiences of mothers of children with bronchiectasis in Counties Manukau, Auckland, New Zealand

**DOI:** 10.1186/s12913-018-3532-9

**Published:** 2018-09-19

**Authors:** Nicola Jepsen, Nadia A. Charania, Sarah Mooney

**Affiliations:** 1 Department of Physiotherapy, Auckland University of Technology, North Campus, 90 Akoranga Drive, Northcote, Auckland, 0627 New Zealand; 2 Department of Public Health, Auckland University of Technology, South Campus, 640 Great South Road, Manukau, Auckland, 2025 New Zealand

**Keywords:** Barriers, Health care access, Bronchiectasis, New Zealand, Child health, Qualitative

## Abstract

**Background:**

Bronchiectasis is a worsening public health problem in New Zealand. This study aimed to explore the health care experiences of mothers of children with bronchiectasis in the Counties Manukau District Health Board area of Auckland, New Zealand.

**Methods:**

Semi-structured interviews were undertaken with ten mothers of children with bronchiectasis. Data were analysed using thematic analysis.

**Results:**

Five themes emerged: 1) Searching for answers, describing mothers’ search for a diagnosis; 2) (Dis)empowerment, describing mothers’ acquisition of knowledge, leading to empowerment; 3) Health care and relationships, describing the impact of relationships on the mother’s health care experiences; 4) A juggling act, describing the challenges of juggling health care with school, work and family; 5) Making it work, describing how mothers overcome barriers to access health care for their child.

**Conclusions:**

The health provider-parent relationship was crucial for fostering positive health care experiences. Mothers’ acquisition of knowledge facilitated empowerment within those relationships. Additionally, mothers’ perceptions of the quality and benefit of health services motivated them to overcome barriers to accessing care. Study findings may help to improve health care experiences for parents of children with bronchiectasis if identified issues are addressed.

**Electronic supplementary material:**

The online version of this article (10.1186/s12913-018-3532-9) contains supplementary material, which is available to authorized users.

## Background

Bronchiectasis is a chronic respiratory disease, characterised by chronic cough and sputum production [1], thought to be caused by a cycle of infection, inflammation and lung damage [1, 2]. Worldwide, rates of bronchiectasis declined in the twentieth century, but bronchiectasis is increasingly recognised as an ongoing problem and cause for concern in developing nations and also in developed nations, particularly amongst indigenous communities [3–5].

In comparison to other developed countries, prevalence of bronchiectasis is high in New Zealand (NZ), where the general population hospitalisation rate increased by 41% between 2000 and 2015 [6]. Of importance are the high rates of bronchiectasis amongst NZ children [7]. Population patterns of bronchiectasis display a significant social gradient in NZ, with Māori, Pacific Island people and those living in lower socioeconomic communities most affected by the disease [6, 8].

Reports of delayed diagnosis of childhood bronchiectasis suggest that there is a limitation in health care access and early identification of signs of infection for children who develop bronchiectasis [7, 9]. By the time children are diagnosed, they have often had a productive cough for some time [10]. A NZ study [7] found that 40% of children in their cohort experienced a productive cough for over two years preceding diagnosis. Access to efficient, effective health care is crucial for identifying respiratory infections early and preventing the development of bronchiectasis. Literature has identified barriers to accessing health care for different populations, including indigenous groups [11–16] and those who experience barriers like transportation and cost [13, 15, 17]. No literature has yet explored health care experiences from the unique perspective of NZ parents of children with bronchiectasis. Research that aims to better understand these experiences may guide improvements to health care services, leading to improved health outcomes for individuals and families through improved understanding of the barriers and enablers to accessing health care, and areas for improvement for health care access in this population.

## Methods

### Research paradigm and methodology

Interpretive description, located in the interpretivist paradigm, guided this study and provided a framework with which to explore people’s experiences of phenomena and develop a practical application of findings [18, 19]. Study findings were also contextualised and framed by the Socio-ecological Model [20].

### Study population

This study was conducted in the Counties Manukau District Health Board (CMDHB) area of Auckland - one of twelve district health boards (DHB) in NZ. Bronchiectasis is a significant problem for the population of CMDHB [6, 21]. The population comprises large Māori and Pacific Island communities and the largest population of children in NZ, many of whom live in poverty [22]. There are more hospital admissions for bronchiectasis in CMDHB than any other DHB in the country [21] and in children, the rate of hospitalisation in CMDHB is just under 60/100,000, while the rate in NZ is less than 30/100,000 [23].

Parents or caregivers of a child (aged 0–17 years) with bronchiectasis were recruited from paediatric and youth clinics at Manukau Super Clinic, CMDHB, and Starship Hospital, which is part of the Auckland DHB. The two DHBs share the care of children with bronchiectasis living in the CMDHB area. Interpreting services were available if required, but were not requested or used for any participants.

### Sampling and recruitment

Purposive sampling aimed to achieve maximum variation in the sample across a range of selected demographic characteristics [18, 24], such as ethnicity, income level, family composition and primary location of care. Demographic information was collected and guided further recruitment. Snowball sampling was also used to recruit participants.

Key clinic staff, including nurses and physiotherapists, helped with recruitment. Flyers, detailing information about the study and the researcher’s contact details, were available in the waiting rooms of clinics for potential participants to access. Patients who did not attend a scheduled appointment were still eligible for recruitment - clinic staff were requested to inform these patients about the study when they phoned the patient to follow up on the missed appointment. No participants were recruited this way. Difficulties with recruitment led to changes to the recruitment strategy to try to ameliorate these difficulties; for example, extending the age of eligible children (from 0-10 years to 0–17 years) and using snowball sampling as an additional sampling method.

### Data collection and analysis

Semi-structured interviews were undertaken by one researcher (NJ), who had no prior relationship with participants and had some qualitative interviewing experience. Semi-structured interviewing allowed researcher flexibility, while providing some guidance around areas of questioning [18, 25]. The interview schedule was based on focus areas identified from previous literature [11, 13–15, 18]. The schedule was piloted with three pilot participants, who were from a similar population. These interviews were not included in data analysis. Feedback was sought on interview content, but no changes were suggested. The researcher adjusted interview questions based on personal reflection [26]. The final interview schedule is detailed in the Additional file 1. Written, informed consent was obtained prior to commencing each interview. Interviews were conducted between October 2016 and December 2017 at a location mutually agreed upon by the participant and researcher. Each interview lasted 30–60 min, was audio recorded and transcribed verbatim by a typist. Each typed transcript was checked for accuracy by the researcher; listening to the audio recording in its entirety allowed the researcher to become more immersed in the data [27].

Thematic analysis was performed by the first author (NJ) to derive themes from the data, using an inductive approach. The initial stage of analysis was performed manually and included reading and exploring the data [28]. The researcher read through each transcript, identifying ideas that were meaningful to the research question and manually applying initial codes to the text. As recommended by Bazeley [28] and Richards [29], the researcher then used mind-mapping to explore the data further and identify commonalities and linkages between ideas. A code book was developed (detailed in Additional file 2), describing codes and nested categories, and detailing the related parameters [28]. Data were then allocated to codes and categories using qualitative data analysis software QSR NVivo, version 11 (QSR International Pty Ltd., Doncaster, Victoria, Australia). Codes and categories were condensed and re-worked until final themes and sub-themes developed. The researcher achieved saturation of themes, whereby new data fitted into the existing framework, no longer eliciting new ideas [28]. This was agreed through peer debriefing with the research team when interviews nine and ten did not produce any new themes or sub-themes.

### Trustworthiness

The researcher used peer debriefing and member checks to improve credibility of the findings [30]. The researcher and co-authors met frequently to review, critique and discuss ideas arising throughout the research process. A short summary of findings was sent via email to all participants for review. Participants were asked to reply by email if they wanted to clarify or change any details, or if they did not agree with the content of the summary. No participants responded, so no changes were made following this process.

Purposive sampling and thick description, giving a detailed account of study processes and using participants’ quotes to illustrate findings, were used to enhance transferability of findings [30]. An audit trail outlined idea development and evolution of interpretations [28], while researcher reflexivity enhanced the credibility of findings [30]. Reflexivity involved researcher self-reflection, appreciating prior knowledge and potential bias throughout the research process [31] – this was documented in a personal research journal and discussed with the research team.

## Results

Ten people were successfully recruited from eighteen participants identified from clinics. Of those who expressed interest and were approached but did not take part in the study, three declined to participate and five were unable to be contacted. Participant characteristics are displayed in Table 1. While potential participants may have been a parent or caregiver of any gender, those recruited were all mothers of children with bronchiectasis. Children’s ages ranged from three to 16 years. Mothers had different levels of experience within the health system, with one child having been diagnosed with bronchiectasis only a month prior to taking part, while others had been receiving health care for bronchiectasis for up to 15 years. Data analysis produced five key themes, described below with sub-themes and illustrative quotes from participants.

**Table 1:** Demographic characteristics of study participants (*n* = 10). Outlines the demographic characteristics of study participants

Characteristic	n (%)
Participant’s age	
18–29	2 (20)
30–39	3 (30)
40–49	3 (30)
50–59	2 (20)
60 and older	0 (0)
Child’s age	
0–4	1 (10)
5–9	4 (40)
10–14	2 (20)
15–17	3 (30)
Ethnicity*	
Māori	3 (30)
Pacific Island	5 (50)
NZ Pākehā	2 (20)
Asian	0 (0)
Other	1 (10)
Family composition	
Couple with children	8 (80)
Solo parent with children	2 (20)
Other	0 (0)
Household composition	
One family household	10 (100)
Two or more family household	0 (0)
Annual household income**	
Less than $25,000	2 (20)
$26,000 - $50,000	3 (30)
$51,000 - $75,000	2 (20)
$76,000 - $100,000	1 (10)
More than $100,000	2 (20)
Number of general practitioner (GP) visits in the past year***	
None	0 (0)
1–3	4 (40)
4–10	4 (40)
More than 10	2 (20)
Number of hospitalisations in the past year	
None	5 (50)
1–2	5 (50)
3–4	0 (0)
5 or more	0 (0)

*Ethnicity reported as multiple responses; therefore, numbers total more than the participant count

**The mean annual household income in NZ is $100,103 for the year ended June 2017 [54]

***The mean annual number of GP visits for children in NZ is 2.5 [55]

### Searching for answers

Participants described a journey of searching for answers about their child’s illness, from first *being in the dark*, through a stage when *no-one listened,* to finally *having an answer, theorising* about causation and eventually *acceptance* of the diagnosis.

#### Being in the dark

The mothers in the study described a stage, early in their experience, of knowing something was wrong with their child but not having a clear diagnosis. Eight out of ten mothers described frequent trips to a general practitioner (GP) or accident and emergency (A&E) clinic. They would often see different doctors, receive multiple courses of antibiotics but not get any closer to a diagnosis.

*At the beginning when I took her 18 times in 32 days, saying that something is wrong with her breathing and he kept telling me “No it’s fine, she looks wonderful” and deep down, ignorantly, I believed him.* (Participant 6)

#### No-one listened

After having no diagnosis for some time (and in some cases being misdiagnosed), participants described voicing their concerns multiple times to doctors, but no-one listened. Mothers wanted to be listened to and treated as experts on their child.

*I know we are not doctors but sometimes… our hunch sometimes… I reckon can be trusted.* (Participant 2)

#### Having an answer

After receiving a diagnosis, participants felt a mix of emotions, including relief, worry, frustration and shock. Three mothers were thankful the diagnosis was not something they perceived to be worse than bronchiectasis.

*They thought he might have cystic fibrosis as well… thank God he doesn’t have that.* (Participant 1)

Despite the frustration that many mothers felt at having waited a long time for diagnosis, some still acknowledged that the medical team had done their best.

*It was… slow to get the answer, I think... They did the best they could do.* (Participant 2)

#### Theorising

In the time following diagnosis of bronchiectasis, many participants wondered about the cause of the disease. This was a reflective process, thinking back to events in the child’s life that may have contributed to the illness. Some mothers thought it may be linked to previous medical conditions. Several mothers considered their own actions, wondering whether their child’s condition might have been different if they had done more physiotherapy (Participant 1) or taken their child to see a specialist earlier (Participants 2, 4 and 8).

#### Acceptance

The final stage in the search for answers was accepting the diagnosis. Participants described learning to deal with the diagnosis and looking to the future. They considered that learning about the disease, having a plan and thinking positively contributed to their acceptance.

### (Dis)empowerment

This theme is made up of several chronological stages, moving from disempowerment and vulnerability to empowerment and shared respect within the health provider-parent relationship. Mothers sometimes achieved empowerment independently of health care providers, and at other times with their assistance, particularly those who provided adequate information.

#### Vulnerability

In the early stages of their child’s diagnosis, participants experienced a sense of vulnerability, not understanding their child’s illness or how to manage it. Mothers felt like they had to trust the doctor, even if they were not sure that the doctor’s plan was the right course of action. Several participants described feeling helpless and being *“led along”* (Participant 5) by the doctor.

*Sometimes you are guided by people because you think you have to be. Cos they’re the doctor and they know better.* (Participant 8)

#### It’s just mum

Many mothers thought that health providers disregarded their concerns, describing being “*fobbed off”* (Participant 2). This resulted in frustration and mothers feeling like they were not being taken seriously or treated as the expert on their child.

*…He prescribed* [my son] *Amoxicillin, and he doesn’t get better from Amoxicillin. So I tried to tell him “that’s not going to work” but he still didn’t listen and gave me Amoxicillin and he still wasn’t better.* (Participant 1).

#### Finding a voice

During this stage, the mothers were gaining knowledge and asking questions, improving their understanding of the disease and its management. Participants became more empowered within the health provider-parent relationship.

*I said to him, “Well I won’t give the antibiotics unless we get it tested to definitely make sure it is a chest infection”.* (Participant 4)

#### It’s a two-way street

A few participants eventually found that they experienced mutual respect between themselves and their health provider. They had gained enough knowledge and confidence that they could discuss their child’s health openly and felt empowered to be a more active caregiver. One mother described a relationship whereby she and the doctors taught each other.

*…As I knew more… I was able to say, “Well… actually, that isn’t the case”. So I would teach them as well.* (Participant 8)

### Health care and relationships

#### Communication

Clear communication was associated with positive health provider-parent relationships. Having information that was tailored to their needs and health providers who were willing to answer questions was important to all participants. Some participants also noted that communication between health services was important.

*They worked together, I would often see their* [email] *correspondence where they would have a yacka behind the scenes to draw up a plan…* (Participant 6)

Poor communication between health providers, when notes were not updated or were not read by health providers, meant that some participants received conflicting, confusing information. Many mothers were frustrated at having to repeatedly explain their child’s background.

#### Familiarity

Identification of a familiar health provider was important. Mothers who had a good relationship with a key person at their GP practice or clinic reported more positive experiences. Some mothers had such a good relationship with their GP that they felt like their GP was *“more like family”* (Participant 3). One participant, in contrast, described how being *too* familiar with her GP was detrimental to their clinical relationship, when the GP was too casual or laughed off her concerns.

#### Going the extra mile

Participants appreciated health providers who went out of their way to ensure that families received the best care possible. Health providers who assisted with parking costs or welfare needs enabled easier access to health care. Some participants explained that their child was given priority at their GP, which made mothers feel like health providers understood their child’s needs and took their child’s wellbeing seriously.

*Sometimes I don’t even have to see the doctor to get a script written up, if they are fully booked and they can’t see him, but he is unwell, they will still do him up a script, they just know.* (Participant 1)

#### Not feeling confident

Some participants described a lack of confidence in health providers who did not seem confident making clinical decisions.

*I don’t really like it when I am at the doctor and they Google, because, you know, I could do that myself.* (Participant 5)

Several participants described contrasting situations in which their doctor had been *too* confident; in hindsight, they could see that the doctor should have sought another opinion. These participants felt less confident in health providers during subsequent visits.

### A juggling act

#### Family impact

The impact of the child’s health needs on the family was substantial. Several participants described the challenges of keeping up with medications, physiotherapy and other health needs; looking after their child had become health care, not just child care. Support from other family members enabled health care access, while mothers who had little family support reported difficulty accessing health care. Two mothers described the negative impact of their child’s illness on other children in the family, who were *“neglected”* (Participant 7) because of the greater demands from the child with bronchiectasis.

#### Juggling work and school

Most participants talked about the difficulty of managing work around accessing health care for their child. One participant described having to tell her colleagues about her child’s illness, so they wouldn’t think she was *“skiving off”* work (Participant 3). Another mother described her fear of returning to work, because her boss may not have been understanding if she needed to take time off to look after her child. It was also difficult for mothers to manage their child’s schooling alongside their health care needs. One participant felt pressure from teachers to ensure her child did not miss too much school, yet also felt pressure from doctors to attend specialist appointments during the day.

*The teacher asked me if I could try and make the appointments after school… the doctor wrote a letter to school… I don’t want to take her out cos I know she has been missing a lot of school… but it’s the right thing to do because she has to go see the specialist…* (Participant 4)

### Making it work

#### Financial enablers and barriers

Many participants described free GP visits as an enabling factor. Additional enablers, like having a community services card or financial assistance from welfare also helped to alleviate costs. Several participants reported that they sometimes did not have enough money to pay for medications or petrol to get to the GP. One participant said that if she could not pay for medications when they were needed, she would save up to get them as soon as she could.

*There are times that are stretching it… especially being on the benefit… I might not have enough money to get it that week but I will get it the next week.* (Participant 4)

#### Time and waiting

Most mothers reported that appointment times and the amount of time spent waiting for appointments were inconveniences that affected their ability or willingness to seek health care. Limited opening hours at GPs made health care access challenging, if parents had to pay for care at the A&E clinic or face long waiting times at the emergency department. Hospital stays also made it challenging for mothers to manage their families, sometimes being required to stay in the hospital, away from their families, for two weeks at a time.

*I had no babysitter, my* [six] *kids and I actually all went up there that day, cos I couldn’t find anyone… we* [were supposed] *to stay in for two weeks just for her meds, but… they had to send us back home.* (Participant 7)

#### Transport and distance

All participants reported that long travel distance was an inconvenience, particularly for those whose main point of care was at Starship Hospital, a significant distance from their home in South Auckland. Three participants suggested that an outreach clinic at Counties Manukau would make it easier for them to attend clinics. Furthermore, two other participants did not drive and reported challenges getting to specialist clinics using public transportation. Despite this, these mothers made sure they found a way to get to appointments, only missing appointments *“once in a blue moon”* (Participant 3).

#### Managing health care services

Many participants reported challenges with navigating the health system – managing different services required for different health problems, working within both public and private systems and linking care between the GP and specialist care. Participants who were managing different types of services reported that it was difficult to maintain continuity of care.

*…Because we have to see Surgical and Bronchiectasis* [services]*, it would be good if we could see them at the same time. But they have already explained to me that they can’t.* (Participant 1)

## Discussion

To the authors’ knowledge, this is the first study to examine the experiences of accessing health care for mothers of children with bronchiectasis in a NZ population. Five main themes illustrated mothers’ experiences of accessing health care for their child with bronchiectasis.

### Parental relationships with health care providers are crucial to the health care experience

Mothers’ relationships with health providers presented as a dominant theme of this study; this was discussed at length by all participants. During initial health care experiences, many mothers described having negative relationships with health providers who did not listen to their concerns. These experiences have been documented elsewhere [15, 32], with parents not feeling welcome and health providers asking the same questions repeatedly, leading parents to feel like they were not being listened to. Mothers in this study also described how they had signalled their concerns to their doctor but had not been believed; they sought health care services many times before the diagnosis. This may be one reason why many NZ children, as reported in local epidemiological studies [7, 9], have had a prolonged wet cough (an average of two years) prior to diagnosis. These findings suggest that health providers are not identifying early symptoms of bronchiectasis, or considering bronchiectasis as a diagnosis, despite concerns voiced by parents.

An effective clinical relationship was described by Ballantyne et al. [17] as family-centred, collaborative and non-judgemental. Collaboration is crucial to an effective relationship, as parents want to feel like they are trusted as an equal partner in the health care interaction [33]. This study highlighted that parents’ relationships with health providers leave a lasting impression and are crucial to their health care experiences. Emphasising patient-centred care [17, 33] by improving collaboration and communication between health provider and parent and between health providers may help to improve families’ health outcomes and experiences. Several participants in this study described the importance of clear communication between themselves and their health care provider, and one participant (Participant 6) described the positive experience of communicating with her doctor via phone and email, allowing collaboration between herself and two specialist doctors. Improved communication may be enhanced by the use of technology, as has been suggested in the literature – by using open notes [34], email communication [35] or electronic information-sharing systems between services [36]. Health literacy must also be considered when identifying appropriate communication channels; improving population health literacy must also be a long-term goal [37–39].

### Mothers’ acquisition of knowledge leads to greater agency and power within the health care experience

Many mothers interviewed for this study described a process of acquiring knowledge about their child’s condition, gaining agency and power in their health care experiences. Power dynamics between health providers and patients have been documented [40]. In the present study, parents described numerous experiences of feeling vulnerable and unable to speak up about their child’s needs when accessing health care. Many health providers are moving from a paternalistic model of health care towards a more patient-centred model of care [33, 41], whereby health care relationships are a partnership, rather than hierarchical. While patient-centred care is desirable, resistance from health providers has been found, due to perceptions of diminished power [40, 42].

All the mothers in this study described the importance of seeking information to better care for their child’s health. Education was seen as an integral part of a doctor’s role, but often limited in terms of detail, attention or time [43, 44]. Patients (and parents) frequently seek their own information, particularly from the internet, to appear more committed to their child’s health; they wanted health providers to listen to their concerns and engage in meaningful discussion about their child’s health [45]. Knowledge acquisition was important to mothers in this study to feel more empowered within the health provider-parent relationship. Power dynamics in health provider-patient relationships are changing as information becomes more readily available to consumers; this may require a shift in the traditional roles of health providers to enable a relationship based on partnership and equality, rather than power [41]. A shift of power from the paternalistic health provider-patient relationship to a relationship built on partnership and shared learning will be crucial to the development of meaningful health care experiences [41, 42, 46].

### Mothers’ appreciation of the worth of health care services will enable access to health care

Mothers in this study described a range of potential barriers to accessing health services, but they also indicated that, knowing their child’s health was important, if the health service was perceived to be worthwhile, they would *“make it work”* (Participant 1). Some barriers to accessing health care were practical difficulties like financial barriers, transportation and lack of social support. While GP visits are free for children under 13 years in NZ [47], some parents described having difficulty buying medication for their child or accessing costly after-hours care. Indirect costs to accessing health care, like taking time off work and transportation costs [14, 17], are also perceived barriers. Having social support, for example from family members, may assist with transportation and child care, enabling health care access [15, 17, 32]. In this study, parents with little social support had difficulty overcoming barriers to accessing health care. Assistance with transportation and child care for other children in the family would go some way to reducing the impact of these barriers.

Mothers of children with bronchiectasis often juggle many competing life demands, as well as the health of their child. Work, school and family responsibilities presented as barriers to accessing health care, making it challenging for parents to get their child to appointments during working hours [15, 17, 48]. Outreach clinics, as have been effective in a rural Australian setting [49], may be an effective solution for parents like those in this study, who may find that closer, more flexible appointments may allow them to schedule health care around work, school and family demands. This was suggested by several participants in the study. Parents in this study expressed that despite facing barriers to accessing health care, if they appreciated the worth of the service, they would find a way to access health services for their child.

### Study strengths and limitations

There are many notable strengths of this study, including creating a space for parents’ stories of experiences of accessing care for their children. Unlike literature that has interviewed only health providers [50] or has used surveys to collect data [51, 52], this study used interviews to capture rich narratives from consumers of health services. This is the first study to examine the experiences of accessing health care for parents of children with bronchiectasis in NZ. This is important in NZ, especially the CMDHB area, considering the worsening rates of bronchiectasis in these areas.

Despite these strengths, some limitations of this study should be noted. While the researcher attempted to recruit parents who did not attend scheduled appointments, none were recruited. This presents ‘elite bias’, whereby the most articulate, easy-to-reach participants will be most likely to participate in a study [53]. This may have led to a sample who experience few barriers to accessing health care, in comparison to those who may have been unable to attend their clinic appointments, which would present a skewed representation of health care experiences. As well, feedback was sought from participants (member-checking) via email, which may not be equally accessible for all participants and may have limited feedback. Only female parents were recruited, limiting the gender diversity of the study. As well as this, a non-parent caregiver may have different experiences to a parent. Greater diversity in gender and family composition may have presented more varied perceptions and experiences of accessing health care. The researcher, of NZ European (Pākehā) ethnicity, only represented one ethnic/cultural background, which may have hindered open discussion about culture. Participants from different cultures may not have been willing to discuss any cultural needs that may have arisen with a Pākehā person, who may be considered to be allied with the Western health system they were discussing.

## Conclusions

Children who are at risk of developing bronchiectasis often come from populations facing significant social inequities. It is crucial that public health efforts target this population, in order to reduce the impact of bronchiectasis on families and the wider health system. This study identified several important discussion points that may develop areas for practice and service delivery to improve health care access for families of children with bronchiectasis. Firstly, a greater emphasis on patient-centred care, particularly on fostering relationships between health care providers and families. Secondly, improving channels of communication between parents and health providers and between services, incorporating the use of technology. Thirdly, addressing practical barriers such as transportation and child care, by providing outreach clinics or transport and child care assistance for some families.

By creating an opportunity for people to share their stories, this study highlighted new insights into the ways that health systems, services and providers are perceived and experienced by consumers. While this study has focussed on the experiences of mothers of children with bronchiectasis in one distinctive NZ DHB, findings may have relevance to other chronic conditions and health care consumers in other regions. This important information can help those working in the health system to improve services and systems and consider their own practices to better cater to the needs of health care users and provide more tailored, equitable health care.

## Additional files

Additional file 1: Interview Schedule. Outlines the questions asked during the interviews. (DOCX 16 kb)

Additional file 2: Code book. Describes the themes and sub-themes from interviews. (DOCX 21 kb)

## Abbreviations

CMDHB: Counties Manukau District Health Board
DHB: District Health Board
NZ: New Zealand
