# Supplementary material for: Health care experiences of mothers of children with bronchiectasis in Counties Manukau, Auckland, New Zealand
Source: BMC Health Serv Res. 2018 Sep 19;18:722. doi: 10.1186/s12913-018-3532-9 (PMC6145180; doi:10.1186/s12913-018-3532-9)
Supplement: Supplementary file 1 — Interview Schedule. Outlines the questions asked during the interviews. (DOCX 16 kb) [file 12913_2018_3532_MOESM1_ESM.docx]

Interview schedule

| Domain | Example questions |
| --- | --- |
| Knowledge & self-management, individual factors | - Please can you start by telling me about what it’s been like for you and your whānau since [child’s name] has had these breathing problems? - Please can you tell me about how [child’s name] came to be diagnosed with bronchiectasis and what was that like for you? - Probe: How was information given to you about the diagnosis? Was that helpful / unhelpful? - How did you find out about how to manage [child’s name]’s breathing problems? - What do you think are the most important things to help [child’s name]’s breathing problems? |
| GP experiences | - Can you tell me about what it’s like for you when you take [child’s name] to the doctor?   - Probe: How has your family doctor been helpful / unhelpful?   - Probe: What makes attending the GP easier/harder? |
| Clinic experiences and other community health experiences | - Can you tell me about the first time you went to the breathing clinic (at Superclinic / Starship) and what that was like for you?   - Probe: How have those clinics been helpful / unhelpful? - You must have had to get medicines for [child’s name] – can you tell me about how you get the medicines? |
| Hospital experiences | - Has [child’s name] ever had to go into hospital for his/her cough? Can you tell me what it was like for you, being in the hospital with [child’s name]? - What impact did that have on your whānau? |
| Participants’ suggested strategies | - If another child in your whānau got bronchiectasis, what would be the most important advice you’d give their mum/dad? - What do you think have been some of the barriers (or difficulties) to accessing health care for [child’s name] and your family? - Based on your experiences with accessing health care services, is there anything that could be done differently that might make it better or more helpful for families of children with bronchiectasis? |
