# Supplementary material for: Health care experiences of mothers of children with bronchiectasis in Counties Manukau, Auckland, New Zealand
Source: BMC Health Serv Res. 2018 Sep 19;18:722. doi: 10.1186/s12913-018-3532-9 (PMC6145180; doi:10.1186/s12913-018-3532-9)
Supplement: Supplementary file 2 — Code book. Describes the themes and sub-themes from interviews. (DOCX 21 kb) [file 12913_2018_3532_MOESM2_ESM.docx]

**Code book**

| **Category** (bold)   - Codes | Description |
| --- | --- |
| **Searching for answers** | Prior to diagnosis, mum is wanting to know what is wrong with her child. Also anything about mum knowing something was wrong - having a hunch. At the time of diagnosis, feelings around finally having an answer |
| - Being in the dark | Knowing something was wrong but having no diagnosis  *Eg. At the beginning when I took her 18 times in 32 days, saying that something is wrong with her breathing and he kept telling me “No it’s fine, she looks wonderful” and deep down, ignorantly I believed him.* (Participant 6) |
| - No one listened | Descriptions of others not seeing / believing symptoms  Eg. *…He would cough every night until he vomited and no one would really believe us there was a real issue..*. *It got to the stage that I wouldn’t give him pamol - I want you to see, you don’t believe he is actually really, really sick.* (Participant 5) |
| - Having an answer | Feelings around the defining moment of diagnosis, including feelings of blame.  *Eg. “Um… Relieved, yip. Although, worryingly worryful…”* (Participant 8). |
| - Theorising | Wondering about a cause - mum thinks of what the cause may be. Events/ situations/ people that may have triggered / caused the illness.  *Eg. She said, “I think your daughter must have got pneumonia when she was little”, which you know, floored us, because we are pretty onto it parents, couldn’t have seen how without really knowing it, um… still to this day don’t understand that…* (Participant 8) |
| - Acceptance | Learning to deal with it, accepting the diagnosis  Eg. *We went through all sorts of stages, you know going in emotional, to thinking she is going to die, to being educated, understanding, taking on their* [health care providers’] *advice.* (Participant 6) |
| **(Dis)empowerment** | Anything about power changes, mum finding her voice and relationships with power at play (e.g. between doctor, teacher, etc). |
| - Vulnerability | Any situation where mum does not feel she knows enough or is brave enough to stand up to the doctor / suggest a different course of action (having to trust the doctor). Also any situation in which mum feels vulnerable.  Eg. *Sometimes you are guided by people because you think you have to be. Cos they’re the doctor and they know better.* (Participant 8) |
| - It’s just mum | Any situation in which mum feels belittled by the doctor (i.e. they do not respect her opinion)  Eg. *I said “if you just read his notes, like what you can see”… He was like “oh no he doesn’t have bronchiectasis; he has chronic lung disease”. I was like “call it whatever you want” … and then he prescribed [my son] Amoxicillin, and he doesn’t get better from Amoxicillin. So I tried to tell him that “that’s not going to work” but he still didn’t listen and gave me Amoxicillin and he still wasn’t better.* (Participant 1). |
| - Finding a voice | Any situation where mum starts to take control of the situation and gains power and confidence  Eg. *I said to him “well I won’t give the antibiotics unless we get it tested to definitely make sure it is a chest infection”. Then he said “alright then”, and then once we get it tested, sometimes it comes out no that there was no bacteria in the sputum… and then I won’t give her the antibiotics.* (Participant 4) |
| - It’s a 2-way street | Any situation in which there seems to be mutual respect between mum and health care professional  Eg. *I explain to them she is a bronchiectasis patient so I tell them she has the chronic lung disease and I tell them what I do during the day with her physio and if she is on orals and that, so I think they are aware.* (Participant 7) |
| **Health care and relationships** | Anything about forming a relationship with a health care professional - both positive and negative |
| - Communication | Anything about communication with health care professionals – positive and negative.  Eg. *They kind of broke it down for me…and explained it to me in that way… I hate reading. Yeah. That’s just me though… I would rather talk to somebody one on one and get it explained to me.* (Participant 1)  *But when we have to go to A&E sometimes, that’s annoying cos it is another brand new group of brand new doctors every time. They can’t access all of his notes. For example, last week when he was unwell, we went to the A&E clinic and the doctor I saw tried to tell me he didn’t have bronchiectasis. I was just like, “Really?” I was like, “Really?!”* (Participant 1) |
| - Familiarity | Importance of being familiar with health professionals; trust.  Eg. *You know, you are so used to one* [doctor] *telling you about your daughter and then you meet a new one, which you are thinking “Does he really know her, is he aware of her illness? Has he read her notes?”* (Participant 7)  Sometimes health professional can be too familiar  Eg. *It’s like they become too familiar, so they become a friend, more than... You know what I mean? Like, yeah… it’s quite casual… cos he had been my Dr since I was 8.* (Participant 5) |
| - Going the extra mile | When health professionals go above and beyond to assist parents to access health care  Eg. [The nurse] *is really good. She will fill out any forms that I need for Work and Income and fax them through. She will do anything to make it a little bit easier.* (Participant 1) |
| - Not feeling confident | Having little confidence in health professionals who do not seem to confident in making clinical decisions  Eg. *I don’t really like it when I am at the doctor and they Google, because you know, I could do that myself.* (Participant 5)  Or when health professionals are too confident and do not seek another opinion  Eg. *I know GPs are a broad range, they are not specialists in a specific area, but I would much prefer that they said “I don’t know.”* (Participant 5) |
| **A Juggling Act** | Trying to juggle multiple things at once and fitting child's health care into that |
| - Family impact | Includes psychological / social impact on child; impact on family dynamics and family relationships  Eg. *It has affected my son. He has basically… he just got pushed out and you know, poor bugger, he brought himself up, I think*. (Participant 6) |
| - Juggling work and school | Any experiences where mum has had to negotiate challenges with the work place or taking time off work; challenges with taking child out of school  Eg. *I am not looking forward to work, I am just worried about her… if she gets sick… if I got a job, what will happen? … I took on the part time role and it suits me, cos during the school holidays I can look after her, so… we will see how it goes.* (Participant 4) |
| **Making it work** | Practical solutions to potential barriers/ you just make it happen somehow |
| - Financial barriers and enablers | Enablers may be community services card, work, etc. Barriers - real or potential  Eg. *There are times that are stretching it… especially being on the benefit… and on the gas-wise… I mean… now and then… I might not have enough money to get it that week but I will get it the next week.* (Participant 4) |
| - Time and waiting | Anything about timing of appointments and things that make it harder or easier. Also finding time to go to the GP, etc. Also the inconvenience of waiting, spending a long time in hospital, etc.  Eg. *The teacher… asked me if I could try and make the appointments after school. So I actually rang to see if I could change it to the afternoon, but then there would be a waiting time again, there would be a waiting time…* (Participant 4) |
| - Transport and distance | Anything about physically getting to appointments  Eg. *If he was really sick I would take him to the doctor by public transport. Sometimes on raining days I found it hard, or if I have the chance I go in the courtesy van.* (Participant 3) |
| - Managing health care services | Anything to do with navigating the health care system  Eg. *…Because we have to see Surgical and Bronchiectasis* [services]*, it would be good if we could see them at the same time. But they have already explained to me that they can’t.* (Participant 1) |
